# Organosilicone double-long-chain diquaternary ammonium salt acts as a biofilm scavenger to ameliorate colitis induced by dextran sulfate sodium salt

**DOI:** 10.3389/fimmu.2025.1644433

**Published:** 2025-12-12

**Authors:** Shaopei Shi, Guoxing Tang, Juan Wei, Si Shen, Zhihao Ding, Qin An, Hui Tao, Fangyu Wang

**Affiliations:** 1 Department of Nephrology, Jiangning Hospital Affiliated to Nanjing Medical University, Nanjing, Jiangsu, China; 2 Department of Gastroenterology, Jinling Hospital of Nanjing Medical University/General Hospital of Eastern Theater Command, Nanjing, Jiangsu, China

**Keywords:** ulcerative colitis, bacterial biofilm, organosilicone double-long-chaindiquaternary ammonium salt, treatment, colitis

## Abstract

**Objective:**

The treatment of ulcerative colitis (UC) remains challenging due to limited efficacy and significant side effects. Organosilicone Double-Long-Chain Diquaternary Ammonium Salt (JUC Spray Dressing) exhibits antibacterial, anti-inflammatory, and wound-healing properties. This study aimed to evaluate the therapeutic effects of JUC Spray Dressing in a Dextran Sulfate Sodium Salt (DSS)-induced UC mouse model and explore its potential mechanisms of action.

**Methods:**

A UC model was induced in mice using 3% DSS, followed by JUC Spray Dressing enema treatment. Disease activity index (DAI), histological scores, bacterial biofilms on the intestinal mucosa, and tight junction integrity were assessed. Inflammatory cytokine levels in peripheral blood were measured, and 16S rDNA amplicon sequencing was performed to analyze cecal microbiota composition.

**Results:**

JUC Spray Dressing significantly alleviated UC symptoms and reduced colonic congestion, with no significant difference compared to other treatment groups (P > 0.05). All treatments significantly decreased the expression of inflammatory cytokines in peripheral blood (P < 0.0001), with no significant differences among the groups. Additionally, all treatments effectively reduced biofilm thickness and bacterial abundance, improving intestinal barrier integrity. JUC Spray Dressing inhibited harmful bacteria such as *Bacteroides* spp. without significantly altering overall microbial composition.

**Conclusions:**

JUC Spray Dressing effectively removes intestinal bacterial biofilms, reduces inflammation, and enhances barrier function to alleviate UC symptoms. Its efficacy appeared comparable to conventional treatments, suggesting potential as an alternative therapeutic option; however, the present study did not assess mucosal safety, and dedicated toxicology studies are required to establish safety for intraluminal use.

## 1 Introduction

Inflammatory bowel disease (IBD) is a chronic inflammatory condition of the gastrointestinal tract that encompasses Crohn’s disease (CD) and ulcerative colitis (UC) (1). With rapid industrialization and the increasing westernization of dietary habits in China, the incidence of IBD has been rising annually, making it a prevalent digestive disorder (2). Although the exact pathogenesis of IBD remains unclear, it is believed to involve multiple factors, including genetic predisposition, intestinal barrier dysfunction, and gut microbiota imbalances (3–5). Most patients with IBD suffer from recurrent episodes with incomplete remission. Bacterial biofilms are strongly implicated in the pathogenesis of IBD (6). When bacteria adhere to biotic or abiotic surfaces, they secrete proteins and mucopolysaccharides that facilitate biofilm formation, creating structured microbial communities. Once mature, biofilms release bacterial cells that disseminate to new sites, leading to the formation of additional biofilms and perpetuating chronic infection. Bacteria within biofilms are embedded in an extracellular polymeric substances (EPS) matrix, which serves as a protective barrier, enhancing bacterial resistance to external stressors (7). In patients with IBD, the intestinal barrier is compromised, allowing bacteria to penetrate the mucus layer and directly interact with the intestinal epithelium, thereby promoting biofilm formation and exacerbating inflammation. As such, bacterial biofilms in the intestinal mucosa are detected at higher rates in patients with IBD.

Current treatment options for IBD, including 5-aminosalicylic acid (5-ASA, mesalazine), glucocorticoids, immunosuppressants, and biologic agents, are limited by severe adverse effects and high costs. Consequently, the development of novel therapeutic strategies is urgently needed (8). 5-ASA is an anti-inflammatory drug with a chemical structure similar to that of acetylsalicylic acid. The exact mechanism of 5-ASA against UC is unclear; however, 5-ASA reportedly inhibits the production of prostaglandins and leukotrienes, resulting in anti-inflammatory effects. In addition, 5-ASA can inhibit nuclear factor-kB (NF-κB), regulate PPAR-γ receptors, and inhibit DNA damage to the intestinal mucosa mediated by reactive oxygen species (9).

Oral antibiotics can also improve the course of refractory UC. Antibiotics, such as amoxicillin, metronidazole (MTZ), and ciprofloxacin, are commonly used as adjuvant treatments for IBD. In a randomized multi-center trial in Japan, patients with UC who received combined antibiotic treatment achieved more effective clinical remission and endoscopic healing (10). However, the use of antibiotics upsets the balance between bacteria and fungi in the intestine. Fungi, particularly *Candida albicans*, have a potentially pathogenic effect as a precipitating factor for IBD (11). In addition, the use of broad-spectrum antimicrobials increases the probability of *Clostridium difficile* infection in patients with active disease, which is the most common infectious complication in patients with IBD, and further increases the risk of colectomy (12). Therefore, the identification of therapeutic alternatives to antibiotics is a key research goal.

Several studies have shown that targeted regulation of intestinal bacterial biofilms can alleviate experimental colitis in mice (13, 14). Quaternary ammonium salt (QAS) is a cationic ammonium salt formed when the four hydrogen ions of the ammonium ions are substituted by hydrocarbon groups. QAS is a common germicidal component that is adsorbed on the surface of the thallus, with the hydrophobic group inserted into the lipid layer, which permeabilizes and destroys the cell membrane, eventually disrupting cell metabolism and leading to the death of the fungus. In addition, QAS interferes with the synthesis of nucleic acids and proteins (15). Therefore, we hypothesized that QAS could relieve UC by clearing intestinal bacterial biofilms. JUC Spray Dressing(name of U.S. FDA and CE certifications, while the medical device name in China is Long-acting Antimicrobial Material) is a new Organosilicone Double-Long-Chain Diquaternary Ammonium Salt formulation that benefits from the high hydrophobicity of silicone, which further improves the water solubility of the compound. When JUC Spray Dressing contacts the skin or mucosal surfaces, it forms a double antibacterial layer, including an adhesive layer and a positively charged layer. The adhesive layer ensures that the salt can stick to the surface, while the positive cations interact with the negatively charged cell wall and membrane via electrostatic forces in a destructive manner, resulting in bactericidal effects. A previous study has shown that JUC Spray Dressing can prevent bacterial growth by physically killing various bacteria (16). The product is now widely used in wound treatment and indwelling care. Although JUC Spray Dressing has been proven to be safe and non-toxic for humans, information is lacking regarding its bactericidal effects on intestinal bacteria (17).

Therefore, this study aimed to evaluate the therapeutic effects of JUC Spray Dressing in a DSS-induced mouse model of UC. Additionally, we explored its mechanisms of action by analyzing biofilms via electron microscopy, assessing intestinal barrier function, and examining microbial community dynamics.

Given that ulcerative colitis involves mucosal biofilm formation, we selected a colonic topical delivery model to test a biofilm-targeting agent (JUC Spray Dressing). This approach was intended to maximize local biofilm removal at the disease site while limiting systemic exposure. Nevertheless, gastrointestinal mucosal pharmacokinetics and intraluminal retention of JUC in this setting have not yet been defined and will require dedicated studies.

## 2 Methods

### 2.1 Reagents

DSS (molecular weight: 36,000–50,000 Da) was obtained from MP Biomedicals (Santa Ana, CA, USA). The fecal occult blood detection kit was purchased from the Jiancheng Institute of Biological Engineering (Nanjing, China). Mouse enzyme-linked immunosorbent assay (ELISA) kits for interleukin-6 (IL-6), interleukin-1β (IL-1β), and tumor necrosis factor-alpha (TNF-α) were obtained from R&D Systems (Minneapolis, MN, USA). Chloral hydrate solution was sourced from Legend Biotech (Beijing, China). MTZ (0.5 g/100 mL) was provided by Shijiazhuang Siyao Co. Ltd. (Shijiazhuang, China), and mesalazine (28 g/60 mL) was obtained from Dr. Falk Pharma GmbH (Freiburg im Breisgau, Germany). JUC Spray Dressing was purchased from NMS Technologies Co., Ltd. (Nanjing, China).

### 2.2 Animal experimental design

A small sample size was chosen as this study represents the first *in vivo* evaluation of JUC Spray Dressing, with the primary objective of obtaining preliminary data for subsequent, more complex experimental studies. Male C57BL/6J mice (6–8 weeks old, 20–24 g, n=50) were purchased from Charles River (Zhejiang, China) and housed under constant conditions (20 ± 2 °C, humidity 45 ± 5%, 12-hour light/dark cycle, five mice per cage) with standard rat feed available ad libitum. After acclimation for one week, the mice were randomly divided into five groups (n=10) as follows: normal control group, DSS plus phosphate-buffered saline (DSS+PBS) group, DSS plus JUC Spray Dressing (DSS+JUC) group, DSS plus metronidazole(DSS+MTZ) group, and DSS plus mesalazine (DSS + 5-ASA) group. The 10 mice per treatment group were reared in two cages. Mice were randomly assigned to groups using the RAND() function in Microsoft Excel 2021. The UC mouse model was established with DSS according to a previously described method (18). Briefly, mice were administered fresh 3%(w/v) DSS freely for seven consecutive days, resulting in acute UC. Mice in the normal control group were free to drink sterile water. On day 7, mice received enemas (200 μM/mouse, 0.008 mL/g/day) containing PBS, JUC, MTZ (1 g/L), or 5-ASA every other day (19, 20). Prior to enema administration, mice were fasted for 12 hours and anesthetized via intraperitoneal injection of 10% (v/v) chloral hydrate (50 μM/mouse), which took effect within 5–10 minutes. Enema administrations were performed between 12:30 and 14:30 on each treatment day, and the order of group treatments was randomized to minimize circadian or operator bias. All procedures were standardized across groups to ensure consistency. After the enema, each mouse was suspended by its tail upside down for 2 minutes and then returned to its original cage. During the experiment, changes in body weight, occult blood, diarrhea, and stool consistency were observed and recorded daily and the disease activity was scored. The test was performed between 12.30 pm and 2.30 pm and the testing order was randomized daily,with each animal tested at a different time on each test day. The minimum allowed mouse weight before euthanasia was 15 g. At the end of the experiment (day 12), the mice were euthanized, and the colon tissue 1 cm from the anus was excised and fixed with 4%(w/v) paraformaldehyde for subsequent experiments. Mice were anesthetized via intraperitoneal injection of chloral hydrate at 350 mg/kg. This regimen provides approximately 2 hours of anesthesia with relatively shallow depth and limited muscle relaxation; at high doses, myocardial contractility may be depressed and arrhythmias can occur, so animals were continuously monitored. No procedure-related perforation or premature death occurred. The remaining colon tissue was stored at −80°C. Each animal was evaluated by three different investigators, two of whom were blinded to the treatment group. Animals were included in the study if they underwent successful enema treatment or excluded if insertion of the enema resulted in perforation or if the animal died prematurely, thus preventing the collection of behavioral and histological data. All assessments were performed in a blinded fashion by three independent experimenters; inter−rater reliability was high (κ > 0.8), and discrepancies were resolved by consensus. No mice experienced perforation or premature death related to enema administration in this study; therefore, no animals were excluded due to procedural complications.

### 2.3 Assessment of inflammation

Changes in body weight, DAI, colon length, and histological score were used to evaluate the successful establishment of the UC mouse model and treatment effectiveness. All collected data were included in the analysis. The DAI was calculated as follows: 1) change in weight (0: <1%, 1: 1–5%, 2: 5–10%, 4: >15%), 2) stool consistency (0: normal, 2: loose, 4: diarrhea), and presence of blood in stool (0: negative, 2: positive, 4: gross bleeding). Colon tissue fixed with 4% (w/v) paraformaldehyde was embedded in paraffin, sectioned, and stained with hematoxylin and eosin. The histological conditions were then determined under light microscopy and scored based on crypt damage, ulceration, and neutrophil infiltration. The inflammation score was determined as follows:1) severity of inflammation (0: none; 1: slight; 2: moderate; 3: severe); 2) extent of inflammation (0: none; 1: mucosa; 2: mucosa and submucosa; 3: transmural); 3) extent of damage to the crypt (0: none; 1: basal 1/3 damage; 2: basal 2/3 damage; 3: only the surface epithelium intact; 4: entire crypt and epithelium lost); and 4) percentage of area infiltrated (1: 1–25%; 2: 25–50%; 3: 51–75%; 4: 76–100%). More than three high-power fields (×400) were randomly selected from each tissue section, and scores were assigned by two blinded independent researchers, with the average value taken as the result.

### 2.4 Immunohistochemistry and immunofluorescence

The expression of inflammatory cytokines in the colon was evaluated via immunohistochemical analysis. Endogenous peroxidase activity was inhibited, and tissue sections were incubated with a nonspecific staining blocking reagent. Next, sections were incubated with corresponding primary antibodies and HRP-conjugated secondary antibodies. Images of the slides were obtained using an Nikon E100 optical microscope (Nikon, Tokyo, Japan). A minimum of three fields per sample were randomly selected for imaging under a 40× objective lens, and semi-quantitative protein expression analysis was conducted using ImageJ software. Protein expression intensity was expressed as the area occupied by the positive signal. The expression of tight junction (TJ)-associated proteins in the colon was evaluated using immunofluorescence. Images of the slides were obtained using an ECLIPSE TI-SR fluorescence microscope (Nikon). For IHC, staining was quantified using H−score/average optical density; for immunofluorescence (MUC2, ZO−1, occludin), mean fluorescence intensity was measured. Negative controls (omission of primary antibody) and isotype controls were included to verify staining specificity.

### 2.5 Measurement of goblet cells

After dewaxing and hydration, the paraffinized sections of colon tissue were immersed in distilled water, then dipped in periodic acid solution, maintained at room temperature for 1.5 hours, immersed in distilled water, dipped in Schiff’s reagent, dewatered in an oven at 37 °C for 20 minutes, rinsed with distilled water for 10 minutes, dehydrated step-by-step, made transparent with xylene, and lastly sealed with neutral gum. Goblet cells in the colon stained dark blue, while the surrounding tissue appeared light blue or colorless. Image-pro Plus 6.0 software was used to analyze the sections.

### 2.6 Electron microscopy

The intestinal mucosa of three randomly selected mice in each group was observed by electron microscopy. Colon tissue 1 cm from the anus was fixed using 2.5% glutaraldehyde. Three microscopic fields were examined for each specimen. Biofilms on the intestinal mucosa were examined using an SU8100 scanning electron microscope (SEM; Hitachi, Tokyo, Japan), while tight junctions between epithelial cells were assessed using an HT7700 transmission electron microscope (TEM; Hitachi).

### 2.7 Serum cytokine analysis using ELISA

Serum was assayed for pro-inflammatory cytokines using specific ELISA kits (R&D Systems, Minneapolis, MN, USA) according to the manufacturer’s instructions.

### 2.8 Sequencing analysis of 16S RNA amplicon

Genomic DNA was extracted from the mouse cecal contents using a Mag Pure Soil DNA KF Kit (Angen Biotech, Guangzhou, China), and DNA purity and concentration were detected by agarose gel electrophoresis. Using diluted genomic DNA as a template, polymerase chain reaction amplification and purification were performed using primers for the bacterial 16S rDNA (V3-V4) region (343-5'-TACGGRAGGCAGCAG-3': forward primer, 798R-5'-AGGGTATCTAATCCT-3': reverse primer). Based on sequence alignment, PyNAST software was used to construct the phylogenetic relationship of representative sequences of operational taxonomic units (OTUs). After pre-processing the sequencing data to generate high-quality sequences, the sequences were grouped into multiple OTUs according to their similarity. Finally, the alpha diversity, beta diversity, taxonomy, and flora composition of the microflora were analyzed and 16S functional gene prediction was performed.

### 2.9 Statistical analysis

GraphPad Prism software (GraphPad Software, La Jolla, CA, USA) was used to perform the statistical analysis and graph generation. The Kolmogorov–Smirnov method was used to analyze the data, and P>0.1 was considered indicative of normal distribution. One-way ANOVA was used to compare mean mouse body weight, colon length, and inflammatory factor levels among groups, followed by Tukey’s *post hoc* test for pairwise comparisons. P<0.05 was considered to be a statistically significant difference.

## 3 Results

### 3.1 JUC Spray Dressing ameliorates colitis induced by DSS

**Figure 1:**
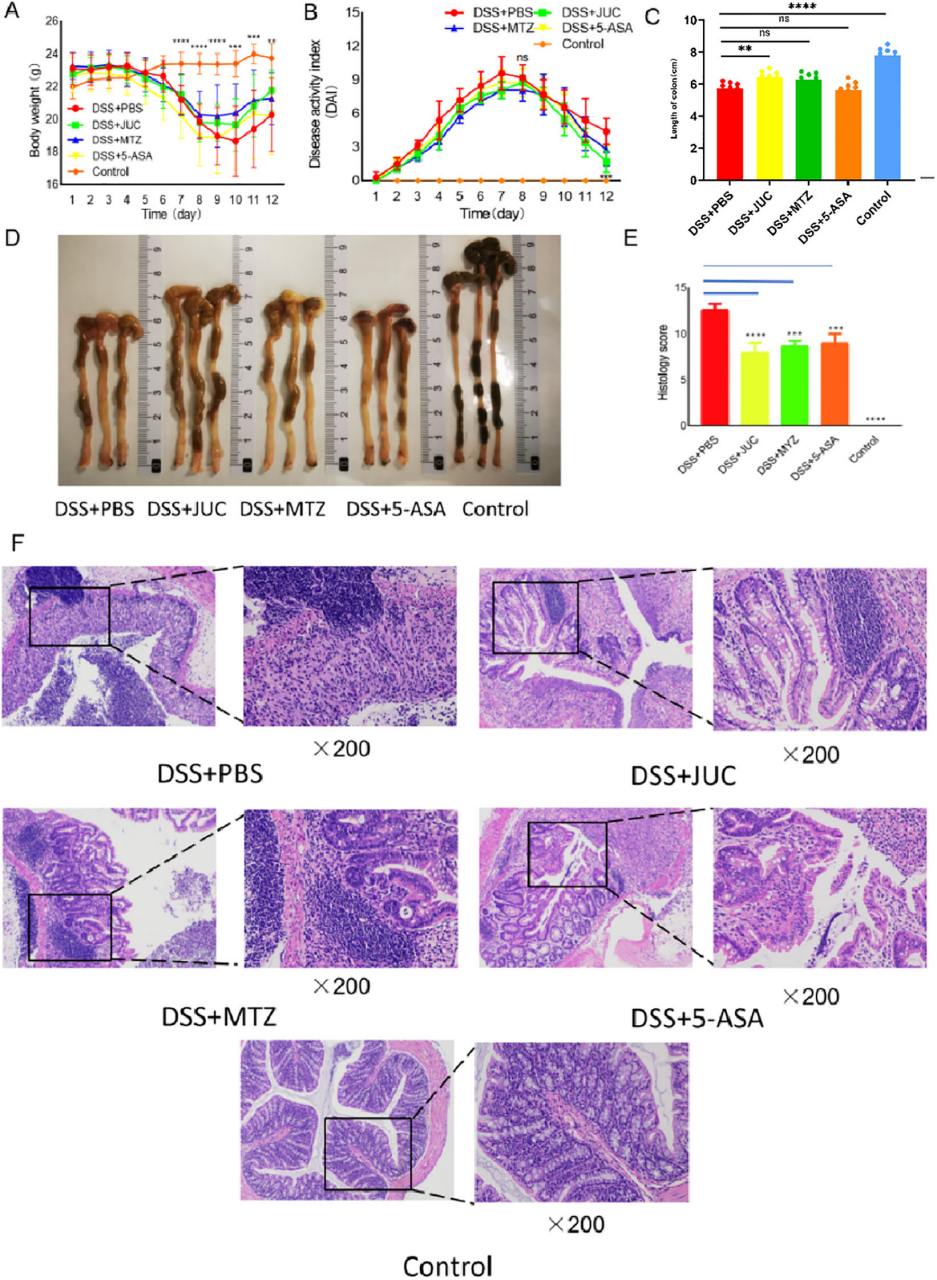
Effects of JUC Spray Dressing on intestinal inflammation in mice. **(A)** Changes in the body weights of mice (N = 10),**P<0.01,***P<0.001,****P<0.0001,compared to the DSS+PBS group. **(B)** Changes in DAI scores (N = 10),***P<0.001,compared to the DSS+PBS group. **(C)** Comparison of colonic lengths between groups (N = 10), *P<0.05,****P<0.0001,compared to the DSS+PBS group. **(D)** Macroscopic view of a mouse colon. **(E)** Histological scores of mouse colon tissues,***P<0.001,****P<0.0001, compared to the DSS+PBS group. **(F)** Effects of drugs on colonic pathological changes in mice(HE,200×). Statistical analysis. Continuous multi-group comparisons (e.g., mouse body weight, panel 1A; colon length, panel 1C): Tukey’s Honestly Significant Difference (HSD) test was used for multiple-comparison correction to rigorously evaluate pairwise differences in group means. Ordinal/score outcomes (e.g., disease activity index, panel 1B; histopathology score, panel 1E): Student’s t-test was applied if the data approximately met normality and homoscedasticity assumptions; otherwise, the non-parametric Mann–Whitney U test was used. A two-sided P < 0.05 was considered statistically significant.

During DSS-induced colitis, mice progressively developed symptoms including weight loss, diarrhea, and hematochezia (bloody stools). Compared to the normal control group, mice in the DSS+PBS group exhibited significantly reduced body weights and increased DAI scores. Following enema treatments, weight loss symptoms improved, and DAI scores significantly decreased (P < 0.01) in all three drug-treated groups. However, no statistically significant differences were observed among these groups (P > 0.05) (**Figures 1A, B**). These results demonstrated that JUC Spray Dressing could alleviate the symptoms of weight loss and decrease activity in mice with UC. Moreover, this effect was comparable to that achieved by MTZ or 5-ASA.

For the time-course endpoints shown in **Figures 1A, B**, the DSS+JUC group did not differ significantly from the DSS+PBS group (p>0.05). Between-treatment differences (JUC vs MTZ vs 5-ASA) likewise did not reach statistical significance.

Mice in the DSS+PBS group showed significant colonic contractures and their intestinal contents were unformed. The experimental results are shown in **Figures 1C, D**. Compared with those in the normal control group, colons in the DSS+PBS group were swollen and hyperemic, and the contractures were shortened (P<0.0001). Colon lengths in the DSS+JUC group were significantly increased (P<0.05) compared to those in the DSS+PBS group. All three drugs were effective in alleviating colon contracture in UC model mice.

Histopathology is an important source of information regarding colon health. As shown in **Figures 1E, F**, the colonic mucosa of the normal control group was intact, the glands were neatly arranged, and no inflammatory edema was observed. In the DSS+PBS group, colon tissue damage was severe, the intestinal mucosa exhibited shedding and necrosis, typical crypt abscess formation was observed, along with glandular deletion, partial crypt damage, and fewer goblet cells, and extensive infiltration of inflammatory cells and transmural inflammation occurred. JUC Spray Dressing, MTZ, and 5-ASA enema therapy alleviated inflammatory cell infiltration and reduced the depth and extent of lesions, thereby reducing the histological score (P<0.001).

### 3.2 JUC Spray Dressing reduces colonic bacterial biofilms

**Figure 2:**
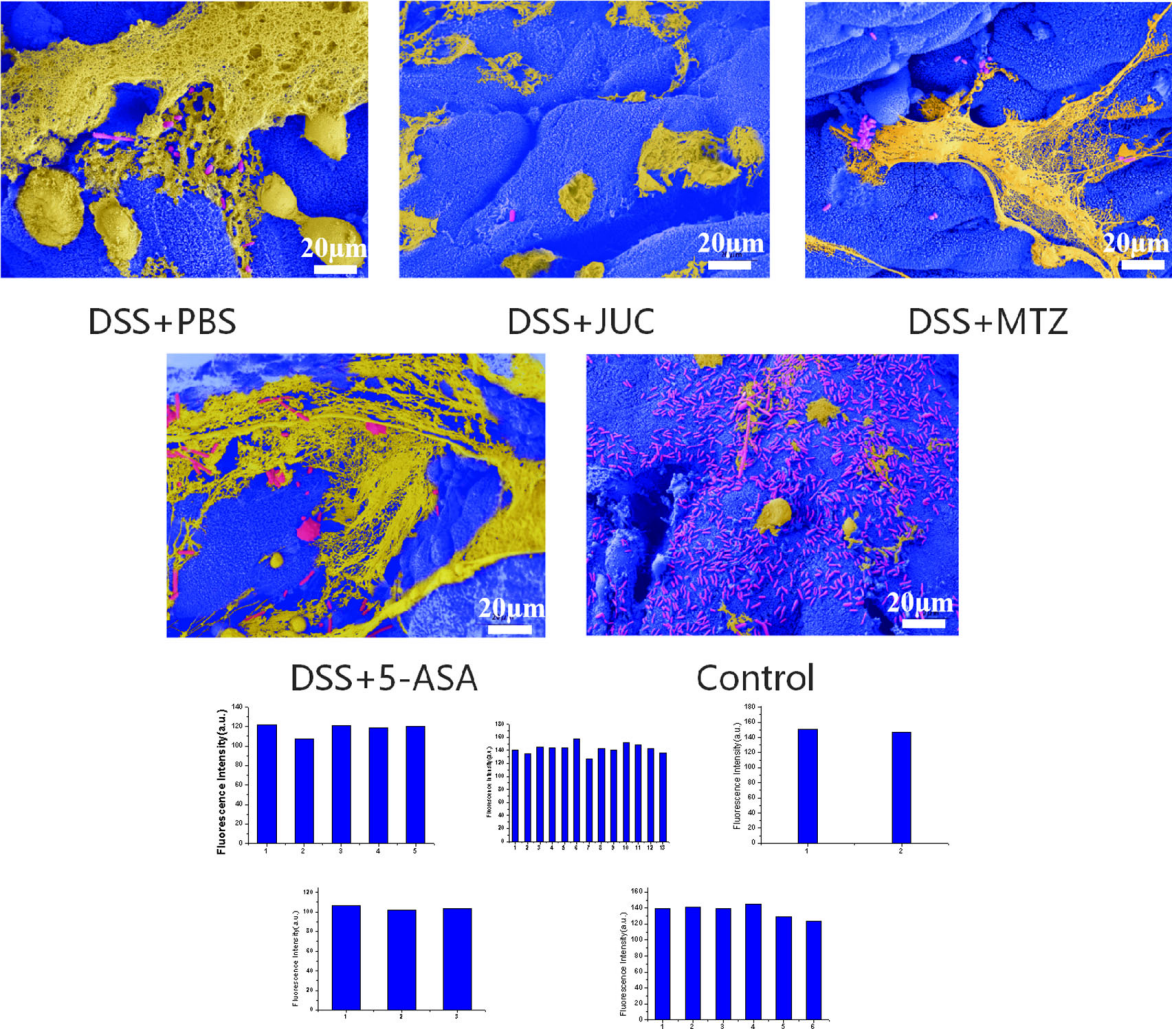
Bacterial biofilms on the intestinal mucosal surface, viewed by scanning electron microscopy(1000×). Quantification of biofilm coverage from SEM images. Biofilm-covered area was measured in ImageJ and expressed as the percentage of mucosal surface occupied by adherent biofilm. Bars show mean ± SD across animals; fields were randomly selected at the same magnification. One-way ANOVA with Tukey’s *post hoc* test was used. Asterisks indicate significance vs DSS+PBS (P<0.05, *P<0.01, **P<0.001); brackets explicitly connect the compared groups.

We performed quantitative analysis of SEM images using ImageJ to calculate the proportion of mucosal surface area covered by biofilm in each group, and compared groups using one−way ANOVA with *post hoc* testing (**Figure 2B**) with consistent magnification and random field selection. The JUC group exhibited a significantly lower biofilm coverage ratio than the DSS+PBS group.

Scanning electron microscopy (SEM) was employed to evaluate the effect of JUC Spray Dressing on intestinal bacterial biofilms. In the DSS+PBS group, SEM revealed biofilms in all 9/9 images. In contrast, biofilm coverage was markedly reduced in 8/9 images from the DSS+JUC group and in 6/9 images from the DSS+MTZ group. However, in the normal control group, the bacteria observed on the surface of the intestinal mucosa were found in planktonic form. As shown in **Figure 2**, biofilm was widely present in the colons of UC model mice and was covered with a thick matrix. In addition to bacteria of various morphologies, many water channels were observed in the biofilms. Unlike in UC model mice, most of the bacteria in the intestinal mucosa of mice in the normal control group were present in planktonic form and biofilm formation was rare. Treatment with MTZ or 5-ASA thinned the EPS matrix and the bacteria were exposed. Treatment with JUC Spray Dressing extensively reduced biofilm formation.

Quantitative SEM analysis (**Figure 2B**) demonstrated a significant reduction in biofilm-covered mucosal area in DSS+JUC versus DSS+PBS, consistent with visual thinning of extracellular polymeric substance (EPS) matrices and exposure of underlying bacteria. Biologically, decreased surface coverage by structured biofilms is expected to lessen epithelial PRR (e.g., TLR2/4) engagement and downstream NF-κB activation, aligning with the observed reductions in IL-6, TNF-α, and IL-1β and the improvements in tight-junction morphology and IF markers (MUC2, ZO-1, occludin).

### 3.3 JUC Spray Dressing decreases inflammatory cytokine production

**Figure 3:**
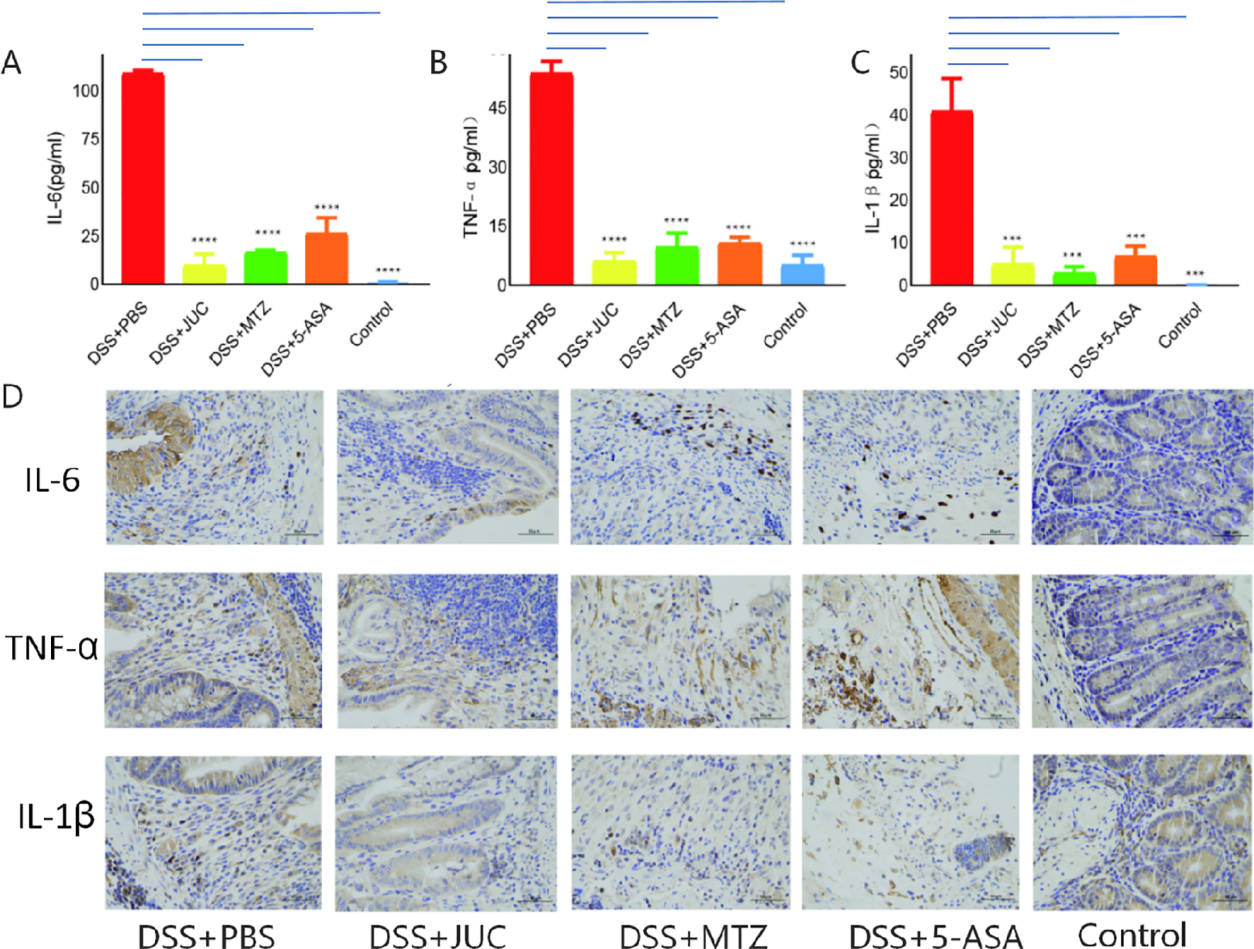
Effects of JUC Spray Dressing on the levels of inflammatory factors in peripheral blood. **(A)** Expression of IL-6 (N = 3). **(B)** Expression of TNF-α(N = 3) **(C)** Expression of IL-1β(N = 3),***P<0.001,****P<0.0001, compared to the DSS+PBS group. **(D)** Immunohistochemical analysis of inflammatory factors in the terminal colon(200×). Statistical analysis. Continuous multi-group comparisons of serum inflammatory cytokine levels (TNF-α, IL-6, IL-1β) were evaluated using Tukey’s Honestly Significant Difference (HSD) test with multiple-comparison correction to assess pairwise differences among groups. A two-sided P < 0.05 was considered statistically significant.

Key pro-inflammatory cytokines associated with UC include IL-6, IL-1β, and TNF-α (21). ELISA was used to measure the level of inflammatory factors in serum and the immunohistochemistry results of end-colon tissue, as shown in **Figure 3**. Levels of IL-6, IL-1β, and TNF-α in the DSS+PBS group were significantly higher than those in the normal control group (P<0.0001). Following treatment with JUC Spray Dressing, MTZ, and 5-ASA enemas, IL-6 levels significantly decreased (P < 0.0001), along with reductions in TNF-α (P < 0.0001) and IL-1β (P < 0.001) levels. The differences among the three treatment groups were not statistically significant (P>0.05).

Quantitative IHC analysis in colon tissue showed trends consistent with serum ELISA, indicating concordant reductions in IL-6, IL-1β, and TNF-α across treatment groups.

### 3.4 JUC Spray Dressing improves intestinal barrier function

**Figure 4:**
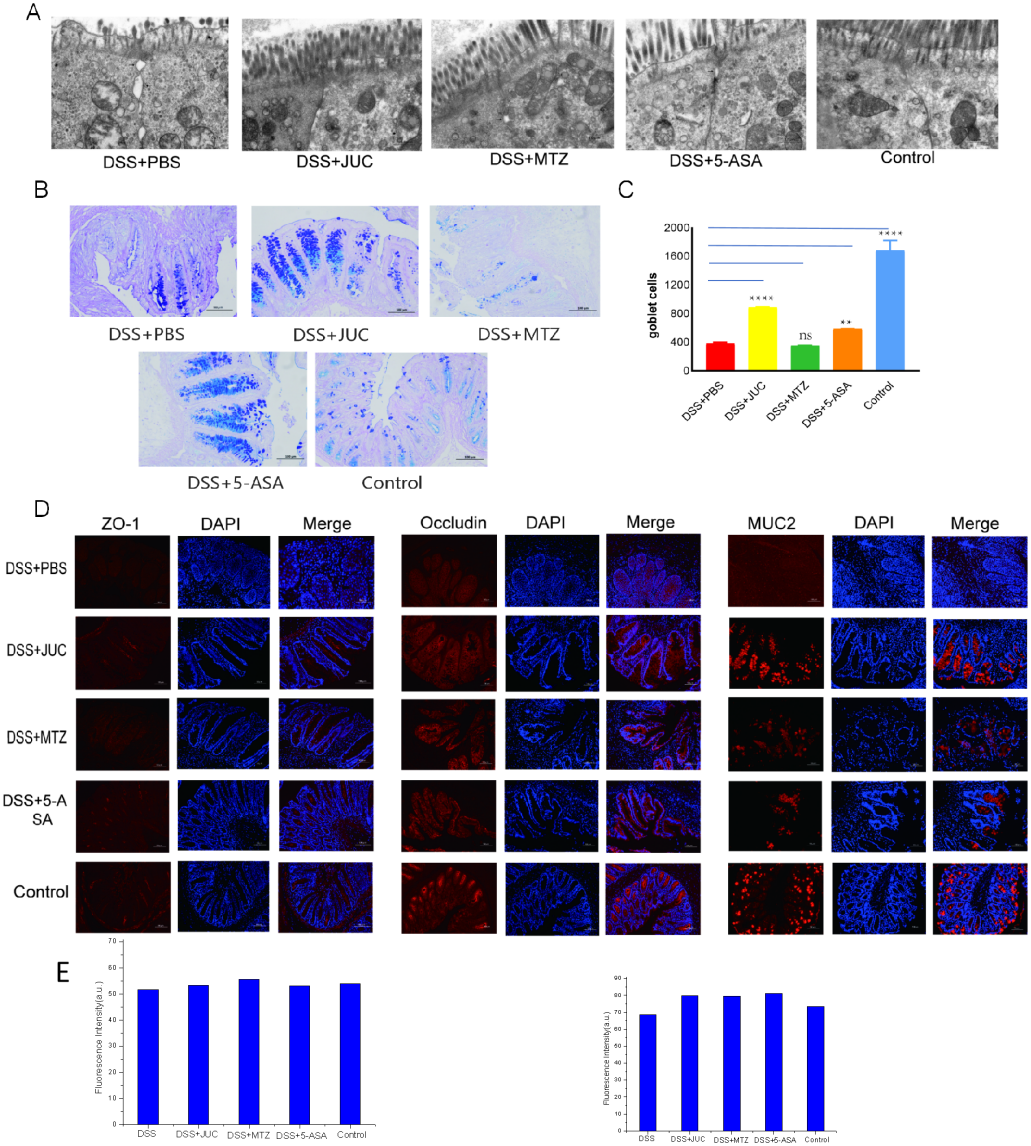
Effects of JUC Spray Dressing on the intestinal barrier function. **(A)** Intestinal mechanical barrier, viewed by transmission electron microscopy. **(B)** Periodic acid-Schiff (PAS)–Alcian blue (AB) stainingof the distal colon(200×). **(C)** Goblet cell count. **(D)** Immunofluorescence analysis of MUC2, ZO-1, occludin(200×). **(E)** Quantification of epithelial barrier markers by immunofluorescence (MUC2, ZO-1, and occludin). Signals were measured as mean fluorescence intensity in ImageJ. Bars show mean ± SD across animals; fields were randomly selected under identical imaging settings. One-way ANOVA with Tukey’s *post hoc* test was used. Asterisks indicate significance vs DSS+PBS (P<0.05, *P<0.01, **P<0.001); brackets explicitly connect the compared groups. Statistical analysis and image quantification. Microscopy images were processed in ImageJ for automated cell counting; counts were based on immunofluorescence-positive cells defined by the specified markers. Continuous multi-group comparisons were evaluated using Tukey’s Honestly Significant Difference (HSD) test with multiple-comparison correction to assess pairwise differences among groups. A two-sided P < 0.05 was considered statistically significant.

After DSS-induced UC, TEM revealed abnormal cell-to-cell connections, including the expansion of apical junction complexes and paracellular spaces. In the DSS+PBS group, the mucus layer was also damaged and thinned under the microscope, the apex was swollen and shedding, the arrangement was complex and sparse, and the organelles were swollen and necrotic. In contrast, the intestinal mucosal chorionic villi in the normal control group were highly consistent and neatly arranged, the organelles were intact, and the cell connections were tight. Treatment with JUC, MTZ, and 5-ASA improved the abnormally tight cell junctions and cytoedema, and relieved barrier structural looseness caused by inflammation-induced TJ protein deletion (**Figure 4A**).

Mucin 2 (MUC2) is a high-molecular-weight glycoprotein secreted by epithelial cells, forming a protective mucus barrier in the intestine (8). After DSS-induced UC, the intestinal barrier of mice was damaged (**Figure 4B**) and the number of goblet cells was reduced (**Figure 4C**). TJs are an important part of the mucosal barrier, preventing harmful substances from leaking out of the intestinal lumen (22). The immunofluorescence analysis of MUC2 and TJ protein levels is shown in **Figure 4D**, the abundance of ZO-1 and occludin-positive cells was reduced in the DSS+PBS group compared to those in the normal control group, and the intestinal barrier function was impaired. The abundance of ZO-1- and occludin-positive cells was increased after JUC Spray Dressing, MTZ, and 5-ASA treatment, and the expression of MUC2 was increased in the JUC and 5-ASA groups, whereas MUC2 expression in the MTZ group was not significantly altered. Quantitative IF confirmed increased ZO−1 and occludin signals in all treatment groups versus DSS+PBS, with MUC2 increased in the JUC and 5−ASA groups and not significantly changed in the MTZ group.

### 3.5 JUC Spray Dressing enhances intestinal microflora composition

**Figure 5:**
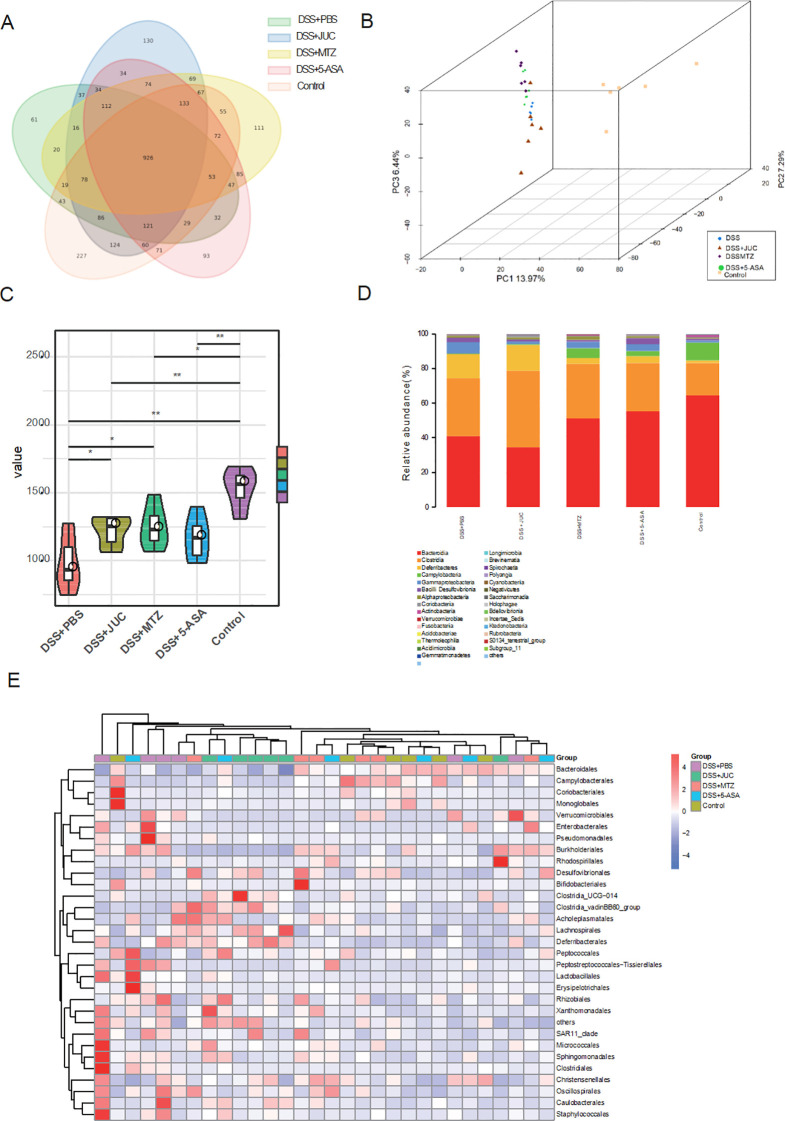
Changes in intestinal flora. **(A)** Venn diagram. **(B)** β diversity analysis. **(C)** α-diversity analysis. *P<0.05,**P<0.01, compared to the DSS+PBS group. **(D)** Histogram of the community structure. **(E)** Heat map.

We assessed α- and β-diversity by 16S rRNA sequencing of cecal contents. JUC increased α-diversity and shifted β-diversity relative to DSS+PBS, whereas phylum-level community composition did not change markedly (**Figure 5**), suggesting restoration of community evenness without wholesale restructuring. This pattern is compatible with selective biofilm disruption and reduced inflammatory drive rather than broad microbiota depletion. Because 16S profiling was performed on cecal contents rather than mucosa-associated communities, biofilm-resident taxa may be under-represented; future studies will sample mucosal layers (e.g., scrapings/biopsies) and apply FISH-based spatial analyses to capture biofilm-associated shifts more directly.

To assess the impact of JUC Spray Dressing on intestinal microbiota composition, 16S rRNA sequencing was conducted on mouse cecal contents, with six samples per group analyzed for operational taxonomic unit (OTU) distribution, as illustrated in the Venn diagram (**Figure 5A**). Overall, 926 OTUs were detected among the five groups, including 61, 130, 111, 93, and 227 OTUs in the DSS+PBS, DSS+JUC, DSS+MTZ, DSS + 5-ASA, and normal control groups, respectively.

Beta (β) diversity is mainly based on the OTU sequence similarity or community structure and is used to compare differences between populations. Intestinal flora diversity of mice was determined using principal coordinate analysis method and heat maps were constructed. Compared with the bacterial community of mice in the normal control group, the bacterial community of mice in the DSS+PBS group exhibited obvious isolation. The β diversity of the microbial community was increased to varying degrees after JUC Spray Dressing, MTZ, or 5-ASA treatment (**Figure 5B**). Alpha (α) diversity can be used to assess species richness and distribution. As shown in **Figure 5C**, the α-diversity correlation violin plot revealed the degree of sample dispersion within each group and the differences in α-diversity between treatment groups. α-Diversity was significantly lower in the DSS+PBS group than in the normal control group. However, α-diversity was significantly increased in the DSS+JUC and DSS+MTZ groups compared to the DSS+PBS group (P < 0.05). The α-diversity of the DSS + 5-ASA group did not differ significantly from that of the DSS+PBS group (P>0.05).

The histogram of the community structure (**Figure 5D**) demonstrated a decrease in bacterial diversity and an increase in the relative abundance of *Clostridium* spp. in the DSS+PBS group compared to those in the normal control group. The community structures did not differ significantly between the DSS+JUC and DSS+PBS groups.

At the phylum level, community composition did not differ significantly between the DSS+JUC and DSS+PBS groups, while α− and β−diversity indices increased after JUC treatment.

Heat maps at the genus level illustrated that probiotics species such as *Roseburia* and *Butyricimonas* were relatively more abundant in the normal control group than in the other groups. The relative abundance of probiotics species was reduced in the DSS+PBS group compared to that in the other groups, whereas the relative abundances of the gengra *Bacteroides and Dorea*, and anaerobic bacteria were higher. Additionally, the relative abundance of anaerobic bacteria was decreased after treatment with JUC Spray Dressing (**Figure 5E**).

Taken together, these data indicate that JUC exerts a selective, biofilm-targeted effect that reduces putatively harmful taxa (e.g., Bacteroides spp.) while maintaining overall community structure, a profile consistent with ecological stability rather than broad microbiota depletion.

## 4 Discussion

This study utilized a DSS-induced mouse model of UC to evaluate the therapeutic effects of JUC Spray Dressing (23). After administration of DSS for 7 days, mice in the DSS group exhibited a significant decrease in activity, weight loss, purulent diarrhea,and bloody stools. Elevated serum levels of inflammatory factors, structural disturbances of the colon glands, shedding and necrosis of the intestinal mucosa, extensive inflammatory cell infiltration, and typical crypt abscess formation demonstrated successful induction of enteritis in mice; thus, the mouse model effectively mimicked clinical UC (24).

Beyond global PRR–NF-κB attenuation, species-specific interactions and cellular targets (e.g., macrophage subsets, Th17 cells) warrant direct testing using gnotobiotic or defined-consortium models and epithelial/immune co-culture systems to delineate molecular mechanisms and specificity.

All three therapeutic agents tested in this study significantly reduced the expression of inflammatory cytokines in DSS-induced colitis, demonstrating their efficacy in mitigating experimental colitis. 5-ASA, which has similar molecular targets as non-steroidal anti-inflammatory drugs (NSAIDs), can inhibit intestinal inflammation and cell proliferation and apoptosis by inhibiting cyclooxygenase(COX) and prostaglandin(PGI). Thus, 5-ASA has been widely used to improve intestinal inflammation and prevent tumorigenesis. Dahl et al. reported that 5-ASA can reduce intracellular polyphosphate levels in many bacteria, making bacteria sensitive to oxygenation stress and thereby reducing colonization and biofilm formation (25). The findings in the current study confirmed that 5-ASA has a certain clearance effect on colon biofilms while improving enteritis in mice. Currently, no studies have reported a direct relationship between QAS, COX, and PGI. Therefore, the exact mechanism by which QAS improves inflammation warrants further investigation. It has been suggested that narrow-spectrum antibiotics in patients with IBD do not achieve the same effect as broad-spectrum antibiotics, suggesting that reducing the colonic bacterial load in patients with IBD may control disease activity (26, 27). According to the results of the current study, both broad-spectrum antimicrobials and physical sterilization with JUC Spray Dressing effectively alleviated experimental colitis in mice by reducing the bacterial load of the colon.

Mechanistically, clearance of biofilms is expected to reduce engagement of epithelial and immune pattern-recognition receptors (PRRs), particularly TLR2/TLR4, thereby dampening NF-κB activation and downstream production of IL-6, TNF-α, and IL-1β. In addition, attenuated TLR/NF-κB signaling may shift mucosal immune balance by limiting pro-inflammatory macrophage activation and constraining Th17 responses; these hypotheses merit direct testing in future work. It also remains unclear whether biofilm formation in UC is a cause or a consequence of mucosal inflammation. These processes may be bidirectionally causal, forming a positive feedback loop in which inflammation facilitates biofilm formation while biofilms further amplify inflammatory signaling; establishing directionality will require longitudinal and interventional studies.

JUC Spray Dressing kills bacteria via electrostatic interactions, thereby minimizing the risk of inducing antimicrobial resistance. A previous study reported that JUC Spray Dressing was effective against a wide variety of microorganisms (17). Acute oral tests in mice showed that JUC Spray Dressing is safe and non-toxic, and JUC Spray Dressing has been widely adopted for skin and wound detoxification and indwelling catheter care (16). These results suggested that JUC Spray Dressing could improve experimental colitis in mice by reducing colonic biofilm formation.

Patients with IBD exhibit increased intestinal permeability, facilitating bacterial translocation and microbial dysbiosis (28). Although it is generally believed that the intestinal flora is involved in the pathogenesis of IBD, most studies have focused on changes in microflora abundance while ignoring the spatial structure of the biofilm (29, 30). Intestinal mucosal biofilms are significantly more abundant in patients with IBD than in healthy subjects, and gut bacteria are more likely to colonize and form biofilms in these patients, leading to inflammation (31, 32). High concentrations of bacteria, similar to those found in biofilms, can lead to high levels of local bacterial antigens, toxins, or other harmful substances (33). Therefore, treatments that target biofilms can improve intestinal inflammation. Meanwhile, intestinal barrier dysfunction is one of the main pathological features of UC (34). Intestinal epithelial cells are covered with mucus secreted by goblet cells, which prevents bacterial invasion (35, 36). TJs between intestinal epithelial cells provide a barrier to the free diffusion of substances and prevent the transposition of intestinal pro-inflammatory substances into the bloodstream (37). The current study findings demonstrated that JUC Spray Dressing can enhance intestinal barrier function, which may be related to its ability to effectively reduce the bacterial load of the colon and promote ulcer healing.

A previous study hypothesized that UC is a disease associated with microbial infections (38). Biofilms formed by large bacterial communities can enhance the penetration of bacteria, destroying the barrier function of the mucus layer. Bacteria come into direct contact with epithelial cells through the sterile mucus layer, causing an abnormal immune response that leads to the development and progression of various diseases (39). A previous study demonstrated that biofilms were found more often in intestinal mucosal biopsy specimens in patients with IBD than healthy individuals (31). In the current study, SEM revealed a larger, richer, and thicker intestinal biofilm in the DSS+PBS group and a variety of channels in the internal structure of the biofilm. Various forms of bacteria can exchange information and nutrients within the biofilm, and a small number of symbiotic bacteria can also form biofilms to enhance interactions with the host (6). Indeed, most of the gut bacteria in the normal control group were present in plank tonic form. However, MTZ or 5-ASA thinned the matrix to varying degrees, exposing the bacteria within the biofilm. Meanwhile, JUC reduced biofilm formation and bacterial abundance over a large area and significantly thinned the matrix, indicating that JUC Spray Dressing can effectively target colonic bacterial biofilms.

Considering the non-specificity of physical sterilization by JUC, 16S rDNA sequencing analysis of mouse ileocecal contents was further performed to explore the effect on intestinal flora composition. The intestinal flora of mice in the DSS+PBS group was dysregulated with low intestinal flora diversity. Meanwhile, the intestinal flora of the DSS+JUC group presented greater α and β diversity than that of the DSS+PBS group, as did that of the DSS+MTZ and DSS + 5-ASA groups. However, the community structure of the intestinal flora was not significantly altered, indicating that JUC Spray Dressing did not significantly affect the composition of the intestinal microbiota. Nevertheless, the content of the cecum,which was not affected by the enema operation, was employed in the current study to analyze the microflora of the mice. Therefore, changes in the mucosal flora should be clarified in future experiments. Overall, compared to MTZ and 5-ASA therapy, which can lead to increased drug resistance, opportunistic infections, and liver and kidney function impairment, JUC Spray Dressing may provide a safe treatment option for UC.

At present, the principles of biofilm intervention mainly include inhibiting adhesion, interfering with the group sensing system among bacteria, promoting biofilm separation (returning bacteria to a planktonic state), destroying the protective screen barrier through mechanical and physical means, and promoting the colonization of probiotics to achieve competitive inhibition (40, 41). As EPS is a protective barrier to biofilm formation, new therapeutic strategies can also inhibit biofilm growth by targeting the destruction of EPS. Surfactants with an electrically charged surface, such as citric acid amphoteric surfactants, can chelate the calcium ion bridge in EPS, making it less stable and easier to destroy (42). In addition, enzymatic digestion by proteases can disrupt biofilms. Patients with IBD have larger mucosal bacteria biofilms and higher iron concentrations within cells than healthy individuals; however, hydrogen sulfide derivatives can remove iron. Motta et al. reported that directly reducing the intake of microbial binders can reduce the ability of IBD-associated mucosal bacteria to form biofilms, without affecting the composition of the bacterial community, thereby improving dinitrobenzene sulfonic acid-induced enteritis in mice (13). The results of the current study also indicated that JUC Spray Dressing reduced biofilm formation without affecting the composition of the bacterial community. However, unlike in previous studies where metal chelating agents destroyed the stability of EPS by directly chelating metal ions, JUC Spray Dressing directly killed bacteria via electrostatic action to destroy the biological membrane. Whether combined use with JUC Spray Dressing can significantly enhance the effects of antibiotic treatment by destroying the protective barrier provided by the biofilm needs to be verified in the future. At the time of writing, obvious drug toxicity has not been reported for JUC Spray Dressing; therefore, further pharmacokinetic experiments are required to determine any potential toxic side effects. Further, whether JUC Spray Dressing is capable of preventing the occurrence of IBD in healthy individuals also warrants further study.

Previous research has linked biofilm formation to the pathogenesis of various diseases, including Barrett’s esophagus, familial adenomatous polyposis, and colorectal cancer (43, 44). Whether targeting biofilms can improve or intervene in the progression of these diseases requires further study. In addition, the anatomy of the mouse intestine differs from that of humans—mice do not have an appendix and the cecum is enlarged—and some studies have proposed that the human appendix plays an important role in the reconstruction of the gut flora after antibiotic use. Thus, while mouse models are extensively utilized in gut microbiome research, replicating the complexity of the human intestinal microbiota remains challenging (45, 46). Finally, while SEM was utilized to visualize mucosal biofilms in this study, future research should incorporate advanced techniques such as three-dimensional (3D) imaging for quantifying biofilm thickness and density, as well as fluorescence *in situ* hybridization (FISH) probes for precise microbial identification.

Long-term implications: although JUC is bactericidal and not intended to increase probiotic taxa (e.g., Roseburia, Butyricimonas), the JUC group exhibited higher α- and β-diversity indices relative to DSS+PBS with only modest phylum-level shifts, consistent with restoration of community evenness rather than wholesale taxonomic restructuring. By selectively disrupting biofilms and reducing inflammatory drive while preserving overall community structure, JUC may lower the inflammatory set-point and strengthen epithelial barrier resilience. In contrast, MTZ can induce broader compositional perturbations and 5-ASA, while anti-inflammatory, does not directly target biofilms. Thus, potential long-term benefit may derive from reduced harmful stimuli and maintained stability rather than direct enrichment of specific probiotic taxa; validation in chronic/relapsing models and functional readouts (e.g., short-chain fatty acid profiling) is warranted. This pattern suggests functional selectivity—targeting biofilm-associated states and inflammatory drive—rather than taxonomic over-pruning. In contrast to MTZ, which often induces broader compositional shifts, JUC’s preservation of overall structure with increased diversity may lower the inflammatory set-point with less risk of dysbiosis over time. Nonetheless, long-term ecological consequences require longitudinal sampling and functional readouts (e.g., short-chain fatty acids, mucus barrier function), ideally including mucosa-associated 16S/shotgun profiling to resolve biofilm-resident species-level selectivity.

Safety and pharmacokinetics: this exploratory study did not include mucosal toxicology, pharmacokinetic, or intraluminal retention analyses for JUC after colonic application. Accordingly, statements regarding safety and local exposure are presented as hypotheses; future work will quantify luminal and tissue concentrations, residence time, and mucosal penetration, alongside formal toxicology.

Limitations: first, the sample size was limited and the study may have been underpowered to detect subtle inter-treatment differences; we therefore used cautious language when comparing JUC with MTZ and 5-ASA. The study was conducted once with n=10 per group, which limits statistical power and external validity; the findings should be viewed as preliminary. Replication in independent cohorts and prespecified power calculations will be incorporated in follow-up studies. Second, we did not perform direct barrier-function assays (e.g., FITC-dextran permeability); our barrier conclusions are based on structural and protein-level readouts and should be validated by functional testing. Third, although we quantified biofilm coverage by image analysis (**Figure 2B**), standardized multicenter pipelines for biofilm metrics would strengthen generalizability.

Outlook: because DSS primarily models acute colitis, future work will evaluate JUC Spray Dressing in chronic and relapsing models to better approximate long−term disease dynamics and therapeutic responses.

## 5 Conclusions

This study evaluated the therapeutic effects of an Organosilicone Double-Long-Chain Diquaternary Ammonium Salt formulation, represented by JUC Spray Dressing, in a DSS-induced mouse model of colitis, comparing its efficacy with conventional treatments such as MTZ and 5-ASA. The results suggest that JUC efficacy is comparable to conventional treatments, suggesting its potential as an alternative therapeutic option for UC, and larger studies are required to validate this comparability. The findings of this study further support the hypothesis that microbial infections contribute to the pathogenesis of UC and that controlling bacterial biofilm formation may alleviate intestinal inflammation. These findings provide valuable insights into the potential clinical applications of Organosilicone Double-Long-Chain Diquaternary Ammonium Salts in UC management.

## Data Availability

The original contributions presented in the study are included in the article/supplementary material. Further inquiries can be directed to the corresponding author.
